# Supplementary material for: A p32 family RNA editing factor acts in mitochondrial ribosome biogenesis
Source: Nucleic Acids Res. 2026 Nov 13;54(21):gkag1169. doi: 10.1093/nar/gkag1169 (PMC13759221; doi:10.1093/nar/gkag1169)
Supplement: Supplementary file 1 [file gkag1169_supplemental_files.zip › Supplementary_data.pdf]

Supplementary Data to:

A p32 family RNA editing factor acts in mitochondrial ribosome biogenesis

Prashant Chauhan[1,2], Ingrid Sveráková-Škodová[1,3,4], Jonathan E. Wong[1,2], Jan Říha[1,2], Julie Dedková[1,2], Anna Huber[1,2], Lukas Müller[1,2], Evgeny S. Gerasimov[5], Alena Zíková[1,2], Vyacheslav Yurchenko[4], Ondřej Gahura[1#]

[1]Institute of Parasitology, Biology Centre of the Czech Academy of Sciences, České Budějovice 37005, Czech Republic
[2]Faculty of Science, University of South Bohemia, České Budějovice 37007, Czech Republic
[3]Department of Biochemistry, Faculty of Natural Sciences, Comenius University, Bratislava 84215, Slovakia
[4]Life Science Research Centre, Faculty of Science, University of Ostrava, Ostrava 71000, Czechia
[5]Faculty of Biology, M.V. Lomonosov Moscow State University, Moscow 199911, Russia
[#]correspondence: gahura@paru.cas.cz

List of Supplementary Data:

Supplementary Figures S1 to S5
Supplementary Table S1: List of oligonucleotides, antibodies, *T. brucei* cell lines, reagents and chemicals, software, instruments and sequences.
Supplementary Table S2: IP-MS data
Supplementary Table S3: Distribution of p32 paralogs in species and higher taxa across eukaryotes including sequences and DeepLoc targeting prediction
Supplementary Data 1: A pdb file with the atomic model of the mt-SSU assemblosome (PDB 9HNY) with the p22 trimer (PDB 3JV1) rigid-body fit into the assemblosome cryoEM map (EMD10180).
Supplementary Data 2: A zip file with sequences of p32 in Euglenozoa and tree files (related to Supplementary Figure S4)

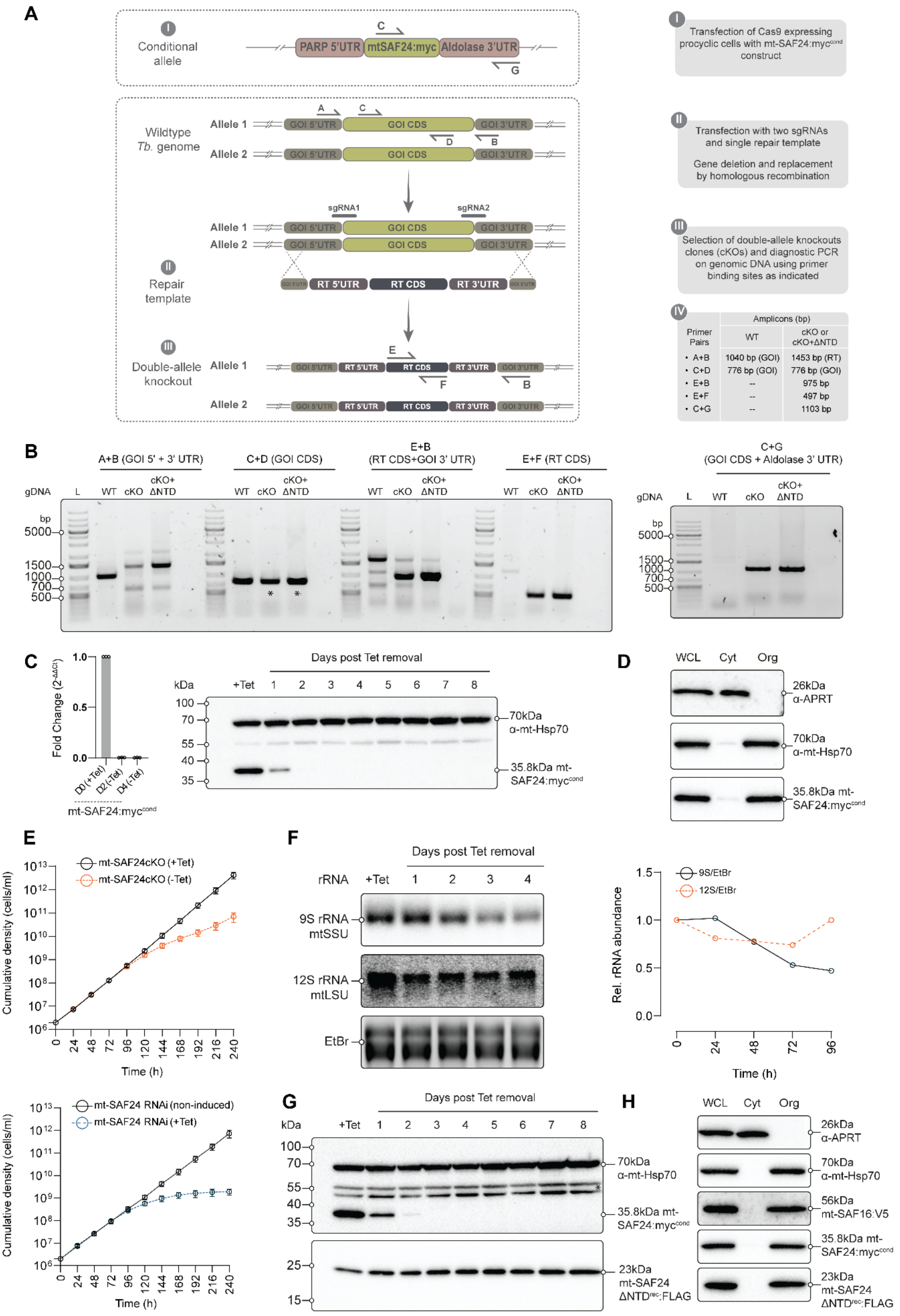

Supplementary Figure S1. Genetic modifications of mtSSU assembly factors and their verifications. (A) A scheme showing genetic modifications to knockout both mt-SAF24 alleles and positions of primers to verify the knock-out and the introduction of a conditional full-length myc-tagged mt-SAF24 (mt-SAF24:myc^{cond}) in the resulting mt-SAF24^{cKO} strain. Lengths of amplicons expected with indicated primer pairs in wild-type (WT) and knock-out (KO) strains are shown. RT; repair template. **(B)** PCR verification of the mt-SAF24^{cKO} strain (KO) and a derived strain co-expressing mt-SAF24-ΔNTD^{rec}:FLAG (KO+ΔNTD). **(C)** Tetracycline-regulated expression of mt-SAF24:myc^{cond} documented on RNA level by q-RT-PCR

(values normalized to cells grown in the presence of tetracycline, β-tubulin gene used as a reference) and on protein level by immunoblotting with anti-myc and anti-mt-Hsp70 (loading control) antibodies. **(D)** Immunoblot of subcellular fractions showing organellar localization of mt-SAF24:myc^{cond} (Wcl - whole cell lysate; Cyt - cytosolic fraction; Org - organellar fraction containing mitochondria). **(E)** Growth curves of the mt-SAF24^{cKO} cell line in presence of tetracycline and after tetracycline removal (top) and the mt-SAF24 RNAi cell with and without RNAi induced (bottom). **(F)** Northern blot analysis of mtSSU (9S) and mtLSU (12S) rRNA levels in the mt-SAF24^{cKO} cells at indicated time points after tetracycline removal. The signal from ethidium bromide-stained gel prior transfer was used as a reference for quantification shown in the right panel. **(G)** Western blot documenting tetracycline-regulated expression of mt-SAF24:myc^{cond} and constitutive expression of mt-SAF24-ΔNTD^{rec}:FLAG with anti-myc, anit-FLAG and anti-mt-Hsp70 (loading control) antibodies. **(H)** Immunoblot of subcellular fractions showing organellar localization of mt-SAF24:myc^{cond}, mt-SAF24-ΔNTD^{rec}:FLAG and mt-SAF16:3×V5.

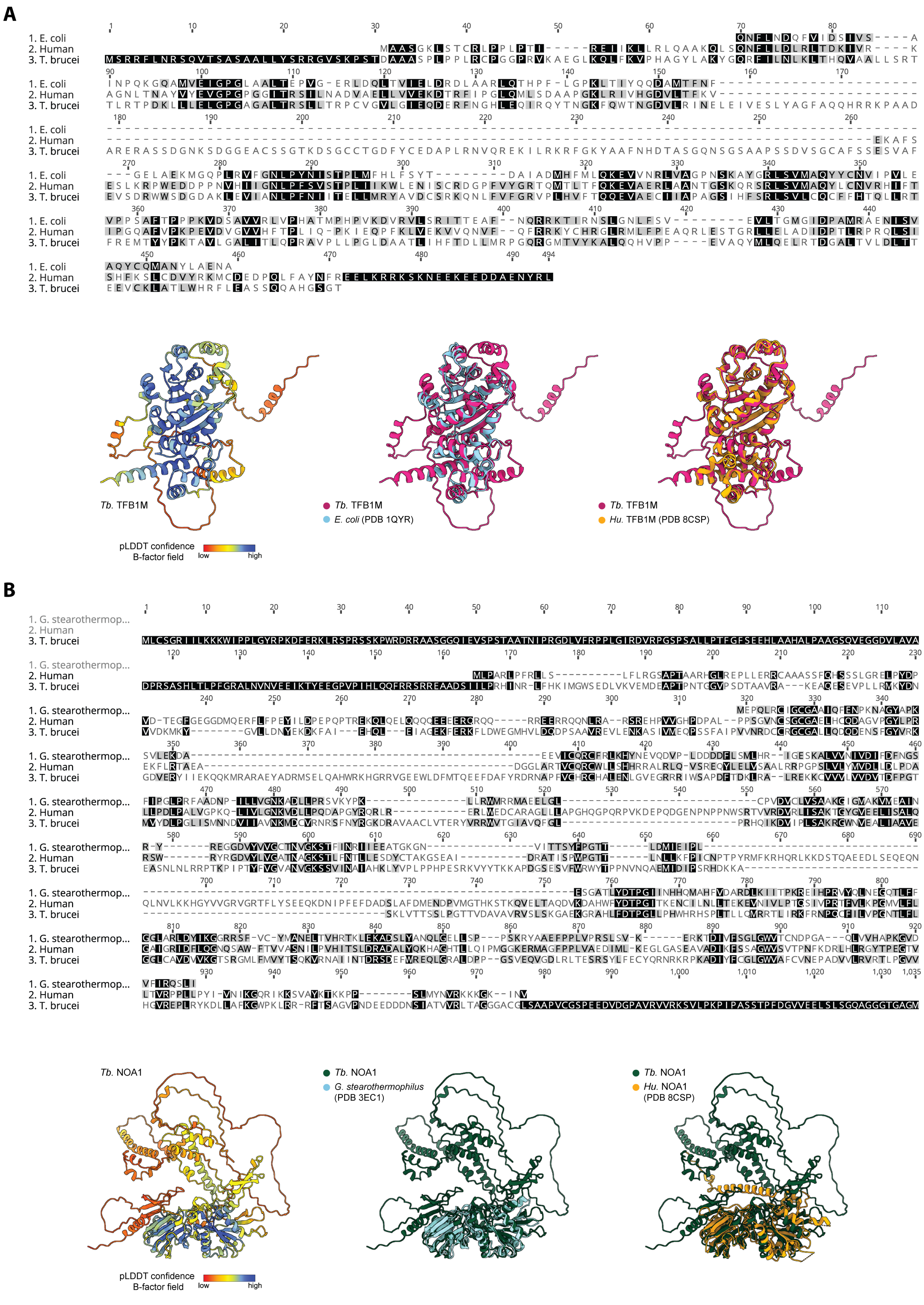

Supplementary Figure S2. *Trypanosoma brucei* encodes conserved assembly factors TFB1M and NOA1. (A) Sequence and structural alignments of *Escherichia coli* KsgA (1), human (2) and *T. brucei* homologs of TFB1M. **(B)** Sequence and structural alignments of *Geobacillus stearothermophilus* YqeH (3), human (2) and *T. brucei* homologs of NOA1. Structures of *T. brucei* proteins were predicted by AlphaFold 3 (4). All other structures were determined experimentally, and PDB accession numbers are included. Sequence alignments were constructed and visualized in Geneious.

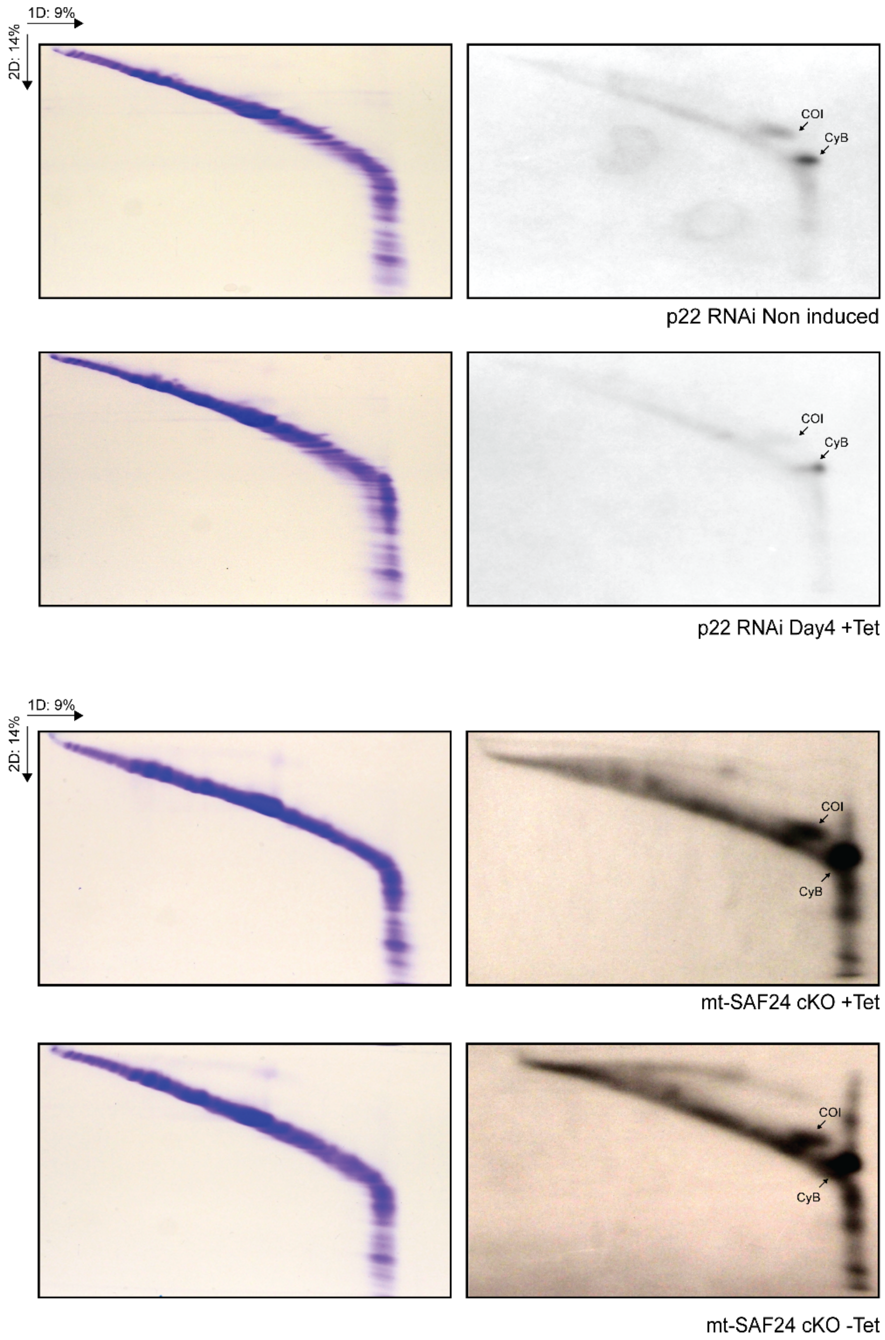

Supplementary Figure S3. Mitochondrial translation efficiency is reduced upon knockdown of p22 and other assembly factors. An independent repeat of the experiment from Fig. 4E. Products of ^{35}S metabolic labelling of products of mitochondrial protein translation from non-induced and tetracycline-induced (4 days) p22 RNAi cells and mt-SAF24^{cKO} strain with and without tetracycline resolved by two-dimensional SDS-PAGE and detected by autoradiography. Coomassie stained gels are shown to document equal loading. Proteins produced by mitoribosome are marked.

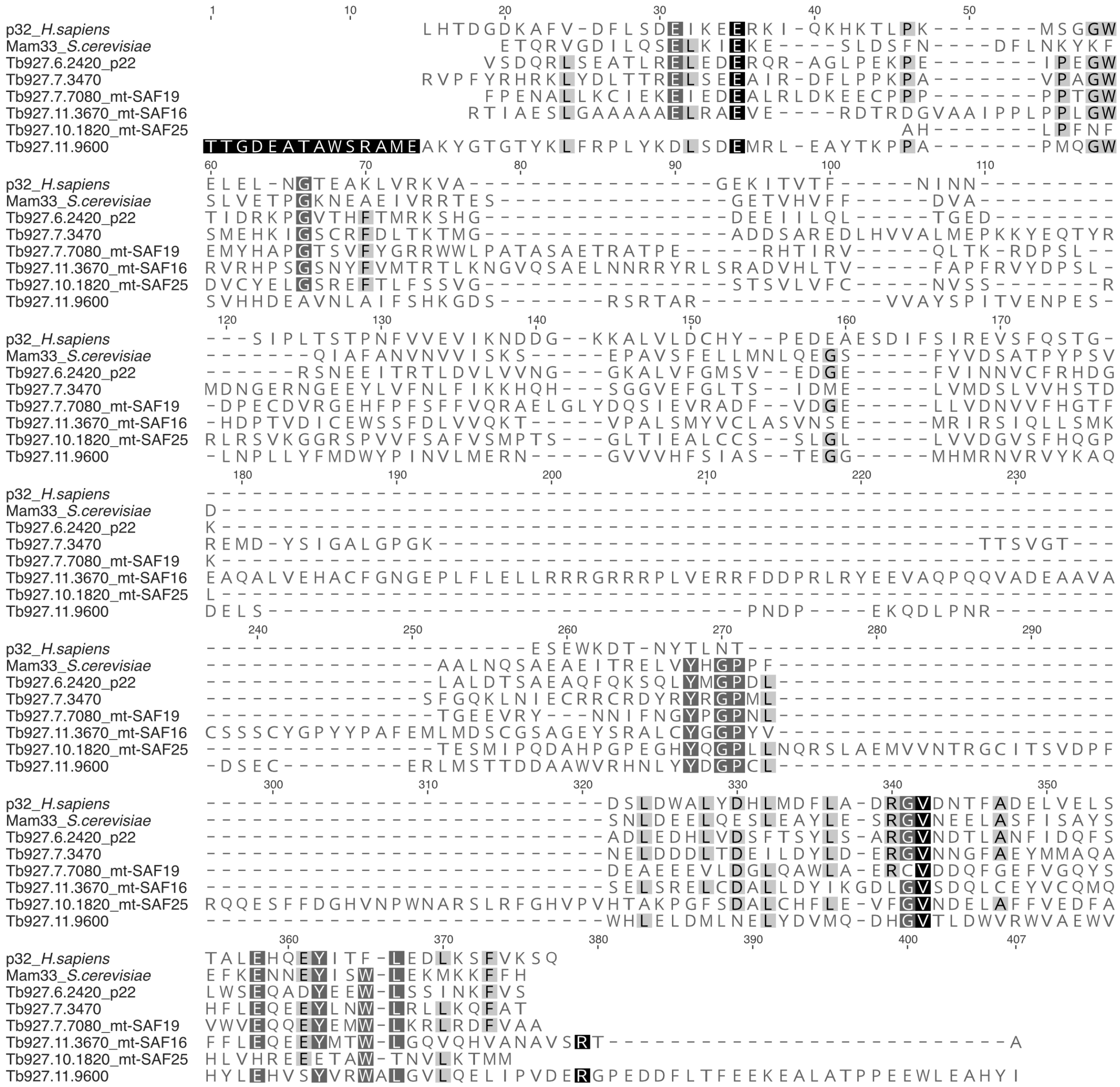

Supplementary Figure S4. Sequence alignment of p32 family proteins. Human p32, Mam33 from *Saccharomyces cerevisiae*, and all six p32 paralogs from *T. brucei* were aligned based on their structures using PROMALS3D, the alignments were visualized in Geneious and adjusted manually. The experimentally detected (p22 (5)) or predicted N-terminal mitochondrial targeting sequences or unstructured regions (all other proteins) were removed. The black, dark grey, and light grey background indicates 100%, <100% and ≥80%, and <80% and ≥60%, identity of residues in given position.

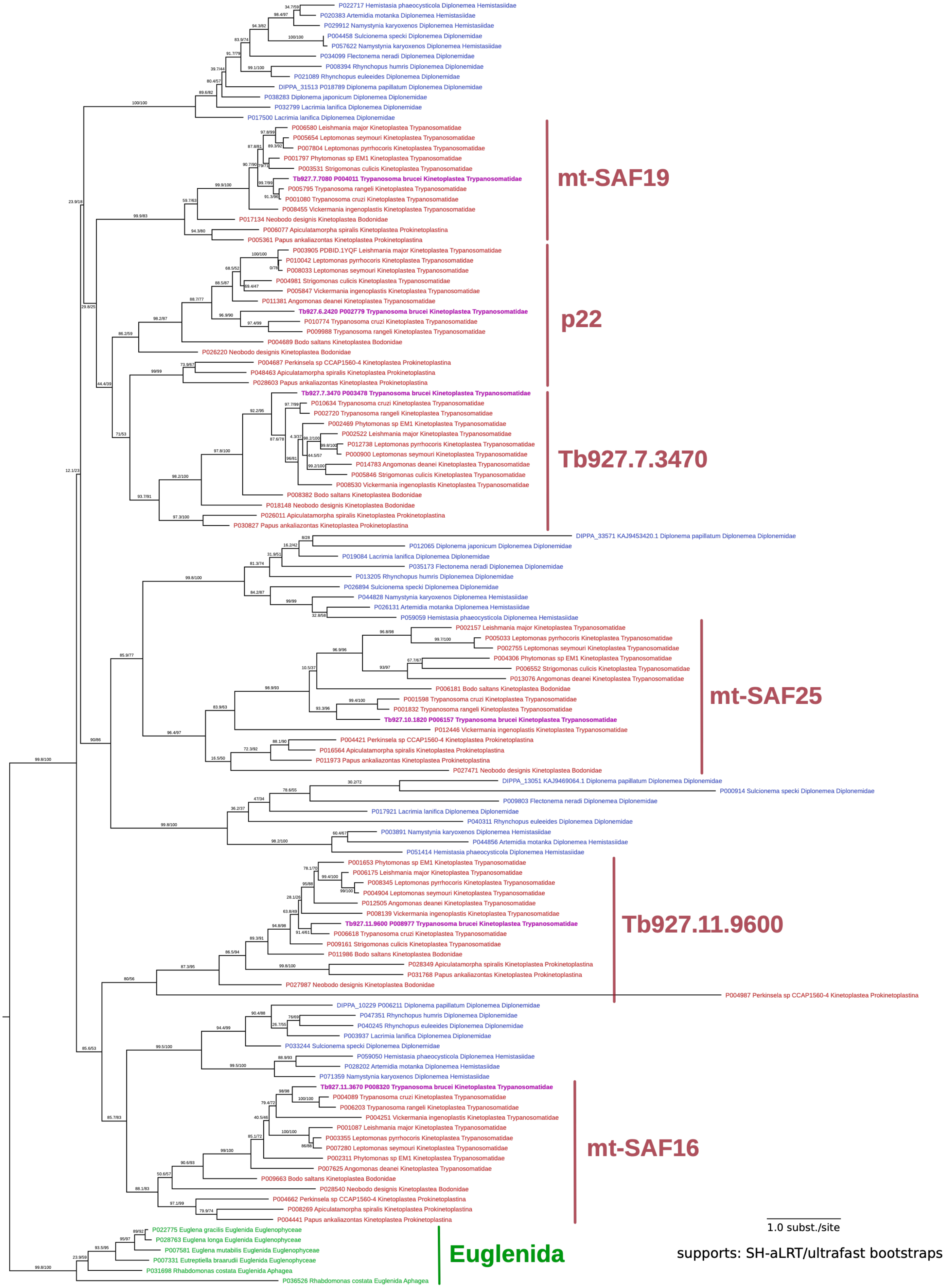

P022717 Hemistasia phaeocysticola Diplonemea Hemistasiidae
P020383 Artemidia motanka Diplonemea Hemistasiidae
P029912 Namystynia karyoxenos Diplonemea Hemistasiidae
P004458 Sulcionema specki Diplonemea Diplonemidae
P057622 Namystynia karyoxenos Diplonemea Hemistasiidae
P034099 Flectonema neradi Diplonemea Diplonemidae
P008394 Rhynchopus humris Diplonemea Diplonemidae
P021089 Rhynchopus euleeides Diplonemea Diplonemidae
DIPPA_31513 P018789 Diplonema papillatum Diplonemea Diplonemidae
P038283 Diplonema japonicum Diplonemea Diplonemidae
P032799 Lacrimia lanifica Diplonemea Diplonemidae
P017500 Lacrimia lanifica Diplonemea Diplonemidae
P006580 Leishmania major Kinetoplastea Trypanosomatidae
P005654 Leptomonas seymouri Kinetoplastea Trypanosomatidae
P007804 Leptomonas pyrrhocoris Kinetoplastea Trypanosomatidae
P001797 Phytomonas sp EM1 Kinetoplastea Trypanosomatidae
P003531 Strigomonas culicis Kinetoplastea Trypanosomatidae
Tb927.7.7080 P004011 Trypanosoma brucei Kinetoplastea Trypanosomatidae
P005795 Trypanosoma rangeli Kinetoplastea Trypanosomatidae
P001080 Trypanosoma cruzi Kinetoplastea Trypanosomatidae
P008455 Vickermania ingenoplastis Kinetoplastea Trypanosomatidae
P017134 Neobodo designis Kinetoplastea Bodonidae
P006077 Apiculatamorpha spiralis Kinetoplastea Prokinetoplastina
P005361 Papus ankaliazontas Kinetoplastea Prokinetoplastina
mt-SAF19
P003905 PDBID.1YQF Leishmania major Kinetoplastea Trypanosomatidae
P010042 Leptomonas pyrrhocoris Kinetoplastea Trypanosomatidae
P008033 Leptomonas seymouri Kinetoplastea Trypanosomatidae
P004981 Strigomonas culicis Kinetoplastea Trypanosomatidae
P005847 Vickermania ingenoplastis Kinetoplastea Trypanosomatidae
P011381 Angomonas deanei Kinetoplastea Trypanosomatidae
Tb927.6.2420 P002779 Trypanosoma brucei Kinetoplastea Trypanosomatidae
P010774 Trypanosoma cruzi Kinetoplastea Trypanosomatidae
P009988 Trypanosoma rangeli Kinetoplastea Trypanosomatidae
P004689 Bodo saltans Kinetoplastea Bodonidae
P026220 Neobodo designis Kinetoplastea Bodonidae
P004687 Perkinsela sp CCAP1560-4 Kinetoplastea Prokinetoplastina
P048463 Apiculatamorpha spiralis Kinetoplastea Prokinetoplastina
P028603 Papus ankaliazontas Kinetoplastea Prokinetoplastina
p22
Tb927.7.3470 P003478 Trypanosoma brucei Kinetoplastea Trypanosomatidae
P010634 Trypanosoma cruzi Kinetoplastea Trypanosomatidae
P002720 Trypanosoma rangeli Kinetoplastea Trypanosomatidae
P002469 Phytomonas sp EM1 Kinetoplastea Trypanosomatidae
P002522 Leishmania major Kinetoplastea Trypanosomatidae
P012738 Leptomonas pyrrhocoris Kinetoplastea Trypanosomatidae
P000900 Leptomonas seymouri Kinetoplastea Trypanosomatidae
P014783 Angomonas deanei Kinetoplastea Trypanosomatidae
P005846 Strigomonas culicis Kinetoplastea Trypanosomatidae
P008530 Vickermania ingenoplastis Kinetoplastea Trypanosomatidae
P008382 Bodo saltans Kinetoplastea Bodonidae
P018148 Neobodo designis Kinetoplastea Bodonidae
P026011 Apiculatamorpha spiralis Kinetoplastea Prokinetoplastina
P030827 Papus ankaliazontas Kinetoplastea Prokinetoplastina
Tb927.7.3470
DIPPA_33571 KAJ9453420.1 Diplonema papillatum Diplonemea Diplonemidae
P012065 Diplonema japonicum Diplonemea Diplonemidae
P019084 Lacrimia lanifica Diplonemea Diplonemidae
P035173 Flectonema neradi Diplonemea Diplonemidae
P013205 Rhynchopus humris Diplonemea Diplonemidae
P026894 Sulcionema specki Diplonemea Diplonemidae
P044828 Namystynia karyoxenos Diplonemea Hemistasiidae
P026131 Artemidia motanka Diplonemea Hemistasiidae
P059059 Hemistasia phaeocysticola Diplonemea Hemistasiidae
P002157 Leishmania major Kinetoplastea Trypanosomatidae
P005033 Leptomonas pyrrhocoris Kinetoplastea Trypanosomatidae
P002755 Leptomonas seymouri Kinetoplastea Trypanosomatidae
P004306 Phytomonas sp EM1 Kinetoplastea Trypanosomatidae
P006552 Strigomonas culicis Kinetoplastea Trypanosomatidae
P013076 Angomonas deanei Kinetoplastea Trypanosomatidae
P006181 Bodo saltans Kinetoplastea Bodonidae
P001598 Trypanosoma cruzi Kinetoplastea Trypanosomatidae
P001832 Trypanosoma rangeli Kinetoplastea Trypanosomatidae
Tb927.10.1820 P006157 Trypanosoma brucei Kinetoplastea Trypanosomatidae
P012446 Vickermania ingenoplastis Kinetoplastea Trypanosomatidae
P004421 Perkinsela sp CCAP1560-4 Kinetoplastea Prokinetoplastina
P016564 Apiculatamorpha spiralis Kinetoplastea Prokinetoplastina
P011973 Papus ankaliazontas Kinetoplastea Prokinetoplastina
P027471 Neobodo designis Kinetoplastea Bodonidae
mt-SAF25
DIPPA_13051 KAJ9469064.1 Diplonema papillatum Diplonemea Diplonemidae
P000914 Sulcionema specki Diplonemea Diplonemidae
P009803 Flectonema neradi Diplonemea Diplonemidae
P017921 Lacrimia lanifica Diplonemea Diplonemidae
P040311 Rhynchopus euleeides Diplonemea Diplonemidae
P003891 Namystynia karyoxenos Diplonemea Hemistasiidae
P044856 Artemidia motanka Diplonemea Hemistasiidae
P051414 Hemistasia phaeocysticola Diplonemea Hemistasiidae
P001653 Phytomonas sp EM1 Kinetoplastea Trypanosomatidae
P006175 Leishmania major Kinetoplastea Trypanosomatidae
P008345 Leptomonas pyrrhocoris Kinetoplastea Trypanosomatidae
P004904 Leptomonas seymouri Kinetoplastea Trypanosomatidae
P012505 Angomonas deanei Kinetoplastea Trypanosomatidae
P008139 Vickermania ingenoplastis Kinetoplastea Trypanosomatidae
Tb927.11.9600 P008977 Trypanosoma brucei Kinetoplastea Trypanosomatidae
P006618 Trypanosoma cruzi Kinetoplastea Trypanosomatidae
P009161 Strigomonas culicis Kinetoplastea Trypanosomatidae
P011986 Bodo saltans Kinetoplastea Bodonidae
P028349 Apiculatamorpha spiralis Kinetoplastea Prokinetoplastina
P031768 Papus ankaliazontas Kinetoplastea Prokinetoplastina
P027987 Neobodo designis Kinetoplastea Bodonidae
P004987 Perkinsela sp CCAP1560-4 Kinetoplastea Prokinetoplastina
Tb927.11.9600
DIPPA_10229 P006211 Diplonema papillatum Diplonemea Diplonemidae
P047351 Rhynchopus humris Diplonemea Diplonemidae
P040245 Rhynchopus euleeides Diplonemea Diplonemidae
P003937 Lacrimia lanifica Diplonemea Diplonemidae
P033244 Sulcionema specki Diplonemea Diplonemidae
P059050 Hemistasia phaeocysticola Diplonemea Hemistasiidae
P028202 Artemidia motanka Diplonemea Hemistasiidae
P071359 Namystynia karyoxenos Diplonemea Hemistasiidae
Tb927.11.3670 P008320 Trypanosoma brucei Kinetoplastea Trypanosomatidae
P004089 Trypanosoma cruzi Kinetoplastea Trypanosomatidae
P006203 Trypanosoma rangeli Kinetoplastea Trypanosomatidae
P004251 Vickermania ingenoplastis Kinetoplastea Trypanosomatidae
P001087 Leishmania major Kinetoplastea Trypanosomatidae
P003355 Leptomonas pyrrhocoris Kinetoplastea Trypanosomatidae
P007280 Leptomonas seymouri Kinetoplastea Trypanosomatidae
P002311 Phytomonas sp EM1 Kinetoplastea Trypanosomatidae
P007625 Angomonas deanei Kinetoplastea Trypanosomatidae
P009663 Bodo saltans Kinetoplastea Bodonidae
P028540 Neobodo designis Kinetoplastea Bodonidae
P004662 Perkinsela sp CCAP1560-4 Kinetoplastea Prokinetoplastina
P008269 Apiculatamorpha spiralis Kinetoplastea Prokinetoplastina
P004441 Papus ankaliazontas Kinetoplastea Prokinetoplastina
mt-SAF16
P022775 Euglena gracilis Euglenida Euglenophyceae
P028763 Euglena longa Euglenida Euglenophyceae
P007581 Euglena mutabilis Euglenida Euglenophyceae
P007331 Eutreptiella braarudii Euglenida Euglenophyceae
P031698 Rhabdomonas costata Euglenida Aphagea
P036526 Rhabdomonas costata Euglenida Aphagea
Euglenida
1.0 subst./site
supports: SH-aLRT/ultrafast bootstraps

Supplementary Figure S5: Phylogenetic tree of p32 proteins from Euglenozoa. The branches corresponding to orthologs of individual *T. brucei* p32 proteins in kinetoplastids and the branch of the single p32 protein in euglenids are indicated. The remaining proteins (shown in blue) are from diplonemids. The tree was inferred by maximum-likelihood analysis using IQ-TREE under the LG+C60+G4 model. Branch support was assessed using 1000 ultrafast bootstrap replicates with the -bnni option and 1000 SH-aLRT replicates. The dataset comprised 126 protein sequences and 395 amino acid positions after trimming. The tree was visualized in FigTree v1.4.4 (https://tree.bio.ed.ac.uk/software/figtree/).

Supplementary References

1. O'Farrell, H.C., Scarsdale, J.N. and Rife, J.P. (2004) Crystal structure of KsgA, a universally conserved rRNA adenine dimethyltransferase in Escherichia coli. *J Mol Biol*, **339**, 337-353.
2. Harper, N.J., Burnside, C. and Klinge, S. (2023) Principles of mitoribosomal small subunit assembly in eukaryotes. *Nature*, **614**, 175-181.
3. Sudhamsu, J., Lee, G.I., Klessig, D.F. and Crane, B.R. (2008) The structure of YqeH. An AtNOS1/AtNOA1 ortholog that couples GTP hydrolysis to molecular recognition. *J Biol Chem*, **283**, 32968-32976.
4. Abramson, J., Adler, J., Dunger, J., Evans, R., Green, T., Pritzel, A., Ronneberger, O., Willmore, L., Ballard, A.J., Bambrick, J. *et al.* (2024) Accurate structure prediction of biomolecular interactions with AlphaFold 3. *Nature*, **630**, 493-500.
5. Hayman, M.L., Miller, M.M., Chandler, D.M., Goulah, C.C. and Read, L.K. (2001) The trypanosome homolog of human p32 interacts with RBP16 and stimulates its gRNA binding activity. *Nucleic Acids Res*, **29**, 5216-5225.
